# Dr. Seymour C. Williamson

**Published:** 1918-07

**Authors:** 


**Dr. Seymour C. Williamson** died in Canisteo May 21, age 80. He graduated from the Baltimore College in 1885.

